# Innovative Safe Sunscreens Technology: Evaluation of Skin Penetration Through In Vitro/In Vivo Assays and Environmental Friendliness

**DOI:** 10.1111/jocd.70538

**Published:** 2025-11-11

**Authors:** Weixiong Huang, Wenwen Zhang, Shota Tomihisa, Hongjuan Kuang, Ru Li, Xueping Chen

**Affiliations:** ^1^ Ausmetics Daily Chemicals (Guangzhou) Co., Ltd. Guangzhou China; ^2^ Glowgenix BioTech Ltd. Hong Kong SAR China; ^3^ Seiwa Kasei Co., Ltd. Higashiosaka Osaka Japan

**Keywords:** Franz cell, microencapsulation, Raman spectroscopy, skin penetration, sunscreen

## Abstract

**Background:**

Conventional sunscreens can penetrate the skin, potentially causing irritation and raising safety concerns. This study introduces a novel sunscreen technology designed to prevent skin penetration while maintaining high efficacy.

**Aims:**

To evaluate the safety, efficacy, and skin penetration profile of an innovative sunscreen that microencapsulated UV filters (octocrylene and avobenzone) within a silk peptide modified polysilicone‐14, and to compare it to a conventional, nonencapsulated formulation.

**Methods:**

The innovative formulation was assessed using a Human Repeated Insult Patch Test (HRIPT) to determine irritation and allergenic potential. Sun protection efficacy was measured in vivo (SPF, PFA). Skin penetration was evaluated using in vitro Franz cell assays with a Strat‐M membrane and in vivo via Raman spectroscopy, which measured penetration into the stratum corneum and epidermis over time. Sensory assessment and tolerability were also conducted on volunteers with sensitive skin.

**Results:**

The HRIPT confirmed the innovative sunscreen was nonirritating and nonallergenic. It demonstrated equivalent sun protection efficacy to the conventional sunscreen, with an SPF of ~30 and PFA of ~10. Crucially, the Franz cell assay showed zero (0.00%) penetration of UV filters for 6 h. Raman spectroscopy confirmed no penetration into the stratum corneum for 4 h and no penetration into the epidermis for 8 h. The formulation was well‐tolerated by sensitive skin volunteers. In contrast, the conventional sunscreen showed significant skin penetration and caused irritation.

**Conclusions:**

The innovative microencapsulation technology successfully creates a safe, non‐skin‐penetrating sunscreen with high UVA/UVB protection. This technology offers a superior safety profile, making it particularly suitable for populations with sensitive skin.

## 1 Introduction

Topical sunscreen application is one of the most effective strategies for preventing sun damage [1]. Although significant advancements have been made in the efficacy and cosmeticity of sunscreens, a major challenge remains in ensuring human safety by preventing skin penetration of UV absorbers into the skin [2, 3]. Clinical studies have confirmed that some sunscreen ingredients can permeate the skin and enter systemic circulation [3, 4, 5, 6], raising concerns about potential adverse effects, including endocrine disruption [7, 8]. This underscores the need for safer UV absorbers and formulation technologies that minimize skin penetration [1, 4].

Sunscreen primarily protects the skin from UV radiation, the primary cause of detrimental sunlight effects. While UVC (100–280 nm) is absorbed by the atmosphere, UVB (280–315 nm) reaches the epidermis, causing surface damage, and UVA (315–400 nm) penetrates deeper into the dermis, leading to DNA damage, sunburn, wrinkles, and photoaging [9]. An ideal sunscreen should combine absorbers effective against both UVB and UVA. Avobenzone is a widely used and effective UVA absorber [9, 10], but it can be photochemically unstable if not properly formulated [9, 10, 11, 12], with degradation products potentially causing photoallergies [5, 13]. Studies show that encapsulating avobenzone, combining it with antioxidants, or formulating it in oil‐in‐water (O/W) emulsions can enhance its stability and reduce skin penetration [4, 6, 9, 11, 14]. Octocrylene, a common UVB absorber, also acts as an excellent stabilizer for other UV absorbers, including avobenzone, improving overall formulation stability [13, 15, 16, 17]. The combination of avobenzone and octocrylene has been reported to provide broad‐spectrum protection with improved photostability and minimal adverse effects [17]. To reduce skin penetration, various formulation strategies have been explored, with encapsulation [17, 18, 19] and O/W emulsification [4] proving effective in stabilizing UV absorbers and limiting their systemic penetration [1, 3, 5].

Multiple methods exist for evaluating the skin permeation of sunscreen ingredients [3]. Common in vitro methods include flow‐through systems, Franz cell diffusion, and tape stripping, while in vivo methods include tape stripping, Raman spectroscopy, and clinical analysis of blood and urine [3]. The Franz cell diffusion system, standardized as OECD TG 428 [20], is widely used in vitro methods employing human skin, pig skin, or synthetic membranes like Strat‐M. Raman spectroscopy, a noninvasive in vivo technique, is gaining popularity for visualizing the spatial distribution of compounds with the skin [21, 22].

This study presents an innovative sunscreen technology that microencapsulates octocrylene and avobenzone using silk peptide polysilicone‐14. We comprehensively evaluated the safety, sun protection efficacy, skin penetration profile, suitability for sensitive skin, and sensory attributes of this innovative formulation in comparison to a general nonencapsulated sunscreen.

## 2 Materials and Methods

### 2.1 Experiment Samples

An innovative sunscreen contained microencapsulated octocrylene and avobenzone (INCI: BUTYL METHOXYDIBENZOYLMETHANE) using silk peptide polysilicone‐14. A conventional sunscreen, formulated without microencapsulation but with minor ingredient adjustments, served as the control. Both samples were O/W emulsions. Detailed formulations are provided in Table 1. To avoid bias, samples were coded prior to analysis.

**TABLE 1:** The innovative sunscreen and general sunscreen formula information.

Innovative sunscreen		General sunscreen	
Ingredient (INCI name)	Content	INCI	Content
AQUA	83.38%	AQUA	83.13%
GLYCERIN	5.00%	GLYCERIN	5.00%
DIMETHICONE	5.00%	DIMETHICONE	5.00%
OCTOCRYLENE	4.10%	OCTOCRYLENE	4.00%
BUTYLOCTYL SALICYLATE	3.00%	BUTYLOCTYL SALICYLATE	3.00%
CETEARYL ALCOHOL	2.40%	CETEARYL ALCOHOL	2.40%
TITANIUM DIOXIDE	1.92%	TITANIUM DIOXIDE	1.92%
ISONONYL ISONONANOATE	1.48%	ISONONYL ISONONANOATE	1.48%
BUTYL METHOXYDIBENZOYLMETHANE	1.03%	BUTYL METHOXYDIBENZOYLMETHANE	1.00%
PENTYLENE GLYCOL	1.00%	PENTYLENE GLYCOL	1.00%
STEARYL ALCOHOL	1.00%	STEARYL ALCOHOL	1.00%
POTASSIUM CETYL PHOSPHATE	1.00%	POTASSIUM CETYL PHOSPHATE	1.00%
POLYSILICONE‐14	0.55%	BUTYROSPERMUM PARKII (SHEA) BUTTER	1.00%
CETEARYL GLUCOSIDE; AMMONIUM ACRYLOYLDIMETHYLTAURATE/VP COPOLYMER; PHENOXYETHANOL; ALUMINA; TOCOPHERYL ACETATE; BUTYLENE GLYCOL; PHENOXYETHANOL; HYDROGEN DIMETHICONE; POLYHYDROXYSTEARIC ACID; DISODIUM EDTA; ETHYLHEXYLGLYCERIN	Each < 1.00%	CETEARYL GLUCOSIDE; AMMONIUM ACRYLOYLDIMETHYLTAURATE/VP COPOLYMER; PHENOXYETHANOL; ALUMINA; TOCOPHERYL ACETATE; HYDROGEN DIMETHICONE; POLYHYDROXYSTEARIC ACID; DISODIUM EDTA; ETHYLHEXYLGLYCERIN; CITRIC ACID	Each < 1.00%

### 2.2 Human Repeated Insult Patch Test (HRIPT)

The HRIPT test was conducted according to the China Safety Technical Specifications for Cosmetics (2015 Edition) [23]. Thirty eligible volunteers (6 males and 24 females; mean age 26.6 ± 6.0 years) who met all the inclusion criteria were enrolled. Approximately 0.020–0.025 g of each sample was applied to a 50‐mm^2^ area on the forearm for 24 h. Skin reactions were assessed at 0.5, 24, and 48 h after patch removal.

### 2.3 In Vivo SPF and PFA Determination

SPF and PFA were determined per the methods described in the China Safety Technical Specifications for Cosmetics (2015 Edition) [23].

#### 2.3.1 SPF Determination

For SPF determination, 10 volunteers (seven males and three females; mean age 36.4 ± 11.9 years), who met the inclusion criteria were recruited. Before testing, the minimum erythema dose (MED) value of the subjects' skin to 290–400 nm UV radiation was determined by exposing five points on their backs to varying dosages of UV radiation. The lowest dosage that caused red spots 24 h postradiation was considered the MED. On the day of testing, a sample of (2.00 ± 0.05) mg/cm^2^ was applied to five spots on the back. The irradiation dosages were selected according to the standard requirements and were carried out under four conditions: (1) Normal skin; (2) Skin coated with a reference substance (prepared according to the high SPF standard); (3) Skin coated with the innovative sunscreen; and (4) Skin coated with the general sunscreen. After 24 h, the MED for the four conditions was recorded, and SPF was calculated as the MED value of protected skin divided by the MED value of unprotected skin. The 95% confidence interval (CI) should not exceed 17% of the mean.

#### 2.3.2 PFA Determination

For PFA determination, 10 volunteers (six males and four females; average age 38.6 ± 6.5 years), who met the inclusion criteria participated. The minimal persistent pigment darkening dose (MPPD) was determined. The test procedures were similar to SPF testing. Before the test, the minimum persistent pigment darkening (MPPD) value of the subjects' skin to 320–400 nm UV radiation was determined and recorded at the end of the test. PFA was calculated as the MPPD value of protected skin divided by the MPPD value of unprotected skin. The 95% confidence interval (CI) should not exceed 17% of the mean.

### 2.4 Skin Penetration Analysis

#### 2.4.1 In Vitro Franz Cell Assay

Percutaneous permeation was evaluated according to OECD TG 428 In Vitro Dermal Absorption Testing [20] and published methods [4]. A Strat‐M membrane (Merck Millipore, Japan) was mounted in a vertical Franz cell apparatus (PermeGear Inc., Hellertown, PA, USA) with the stratum corneum facing up. The receptor compartment contained a 1:1 (v/v) mixture of 20 mM phosphate‐buffered saline (PBS; pH 5.8) and ethanol. After equilibration of the membrane for 30 min to ensure tight contact with the receptor solution, the sunscreen formulations were applied to the membrane surface. The receptor solution was sampled at 2, 4, 6, 8, and 24 h, and the concentrations of octocrylene and avobenzone were quantified using high‐performance liquid chromatography (HPLC) per Chapter 5 of the China Safety Technical Specifications for Cosmetics (2015 Edition) [23].

### 2.5 In Vivo Raman Spectroscopy

Skin penetration was assessed in vivo according to GB/T 40219–2021 General Specification for Raman Spectrometer [24]. A volunteer with a cleaned forearm test area was acclimated to controlled conditions (22°C ± 2°C, 50% ± 10% RH) for 30 min. Each sample was applied at 5 mg/cm^2^ to a 1 × 1 cm^2^ skin area, left for 30 min, and then washed off. At 1, 2, 4, 6, 8, 10, and 12 h post‐application, spectra from 2 points within the test area were collected in parallel five times using a point‐by‐point mapping approach in an x‐z‐coordinate system with a 5 μm depth difference and a 20 × 120 μm scanning area. Measurements were taken using the LabRAM Odyssey (HORIBA) at 2.68 mW laser power and 0.5 s integration time. Raman spectrometry data were analyzed using Labspec (HORIBA) software. Statistics were performed using ORIGIN 2017.

### 2.6 In Vivo Clinical Tests

#### 2.6.1 Sensitive Skin Evaluation

The test followed T/GDCA 029—2023 Evaluation of Cosmetics for Sensitive Skin [25]. Thirty volunteers were selected via a screening questionnaire from healthy adults aged 18–60 with assessed sensitive skin. Inclusion required comprehension of the study, provision of informed consent, and commitment to protocol adherence. Exclusion criteria precluded individuals who were pregnant, lactating, or planning pregnancy; had significant systemic or immunologic disorders; or had received dermatologic treatments or cosmetic procedures on the test area within the preceding 3 months. Further exclusions encompassed recent use of antihistamines (within 1 week), immunosuppressants, retinoids, or corticosteroids (within 1 month); participation in concurrent or recent clinical trials (within 2 months); involuntary enrollment; or active inflammatory skin diseases. Withdrawal criteria encompassed protocol deviations, poor compliance, voluntary withdrawal, significant adverse events, or investigator judgment. Throughout the study, participants were restricted to using the provided test product on the designated area while maintaining consistent habits for all other personal care products, diet, and lifestyle. Subjects applied the sunscreen formulations to each side of their faces once per day for 4 weeks. Subjects were interviewed on days 7, 14, 21, and 28 to record adverse reactions (itching, redness, burning, and stinging).

#### 2.6.2 Sensory Evaluation

Sensory evaluation was performed according to T/GDCA 003—2020 General Rules for Sensory Evaluation of Cosmetics [26]. Twenty volunteers who met the standard requirements acclimated to controlled conditions (22°C ± 2°C, 50% ± 10% RH) for at least 10 mins. They then applied 0.2 mL of the sunscreen formulations to each half of their faces by rolling 10 times. Attributes including spreadability and scrubbiness during application, as well as stickiness, refreshing feeling, residue, nourishment, softness, shininess, and skin naturalness at 2 min post‐application, were evaluated. Each attribute was rated on a scale of 1–5, where 1 indicated “very disagree,” 2 “disagree,” 3 “neutral,” 4 “agree,” and 5 “very agree.”

#### 2.6.3 Statistical Analysis

The results were expressed as mean ± SEM. Intergroup comparisons were performed using a two‐tailed *t*‐test and Wilcoxon signed‐rank test. A *p*‐value < 0.05 was considered statistically significant.

## 3 Results

### 3.1 HRIPT Test

No adverse reactions were observed in any of the volunteers for either sunscreen sample. These results indicate that both formulations were mild and nonirritating to normal human skin under the test conditions.

### 3.2 In Vivo Sun Protection Efficacy (SPF and PFA)

The results of the in vivo clinical SPF and PFA tests were summarized in Table 2. Both the innovative and general sunscreens demonstrated comparable sun protection efficacy, with SPF values of approximately 30 and PFA values of approximately 10, resulting in an SPF/PFA ratio of < 3 for both formulations. The absence of a significant difference in SPF and PFA values indicates that the microencapsulation technology used in the innovative sunscreen did not compromise its sun protection performance.

**TABLE 2:** The in vivo clinical test measured SPF and PFA values.

Sample	SPF (mean ± SEM; 95% CI)	PFA (mean ± SEM; 95% CI)	SPF/PFA ratio
Innovative sunscreen	30.2 ± 0.3; [29.6, 30.9]	10.4 ± 0.2; [9.9, 10.9]	< 3
General sunscreen	31.0 ± 0.2; [30.5, 31.6]	11.6 ± 0.2; [9.0, 10.2]	< 3

### 3.3 Skin Penetration Analysis

#### 3.3.1 In Vitro Franz Cell Assay

**FIGURE 1:**
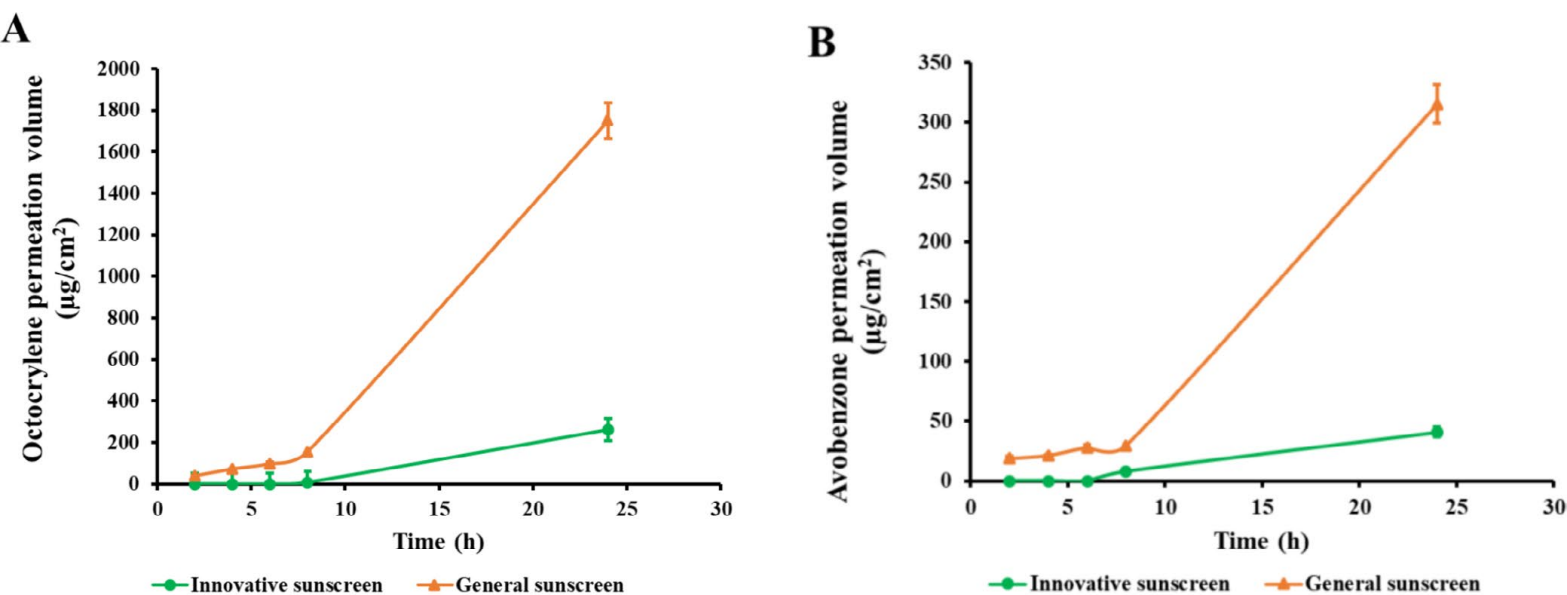
The cumulative amount of octocrylene (A) and avobenzone (B) permeated through the Strat‐M membrane.

The percutaneous permeation of octocrylene and avobenzone was evaluated using a Strat‐M membrane in a Franz cell system. The results, quantified via HPLC analysis (Tables 3 and 4, Figure 1), revealed a stark contrast between the two formulations.

**TABLE 3:** The cumulative amount of octocrylene permeated through the Strat‐M membrane.

Weight (μg/cm^2^) (%)	2 h	4 h	6 h	8 h	24 h
Innovative sunscreen	0 (0.00%)	0 (0.00%)	0 (0.00%)	8.1 ± 0.032 (0.04%)	262 ± 7.85 (1.44%)
General sunscreen	38.9 ± 3.11 (0.14%)	71.8 ± 2.15 (0.25%)	97.5 ± 6.83 (0.34%)	154.6 ± 1.55 (0.54%)	1749 ± 87.47 (6.08%)

**TABLE 4:** The cumulative amount of avobenzone permeated through the Strat‐M membrane.

Weight (μg/cm^2^) (%)	2 h	4 h	6 h	8 h	24 h
Innovative sunscreen	0 (0.00%)	0 (0.00%)	0 (0.00%)	8 ± 0.032 (0.17%)	41 ± 4.10 (0.90%)
General sunscreen	18.8 ± 1.50 (0.25%)	21.4 ± 0.64 (0.29%)	28 ± 1.96 (0.37%)	29.8 ± 0.30 (0.40%)	316 ± 15.78 (4.20%)

For the innovative sunscreen, neither octocrylene (Table 3) nor avobenzone (Table 4) was detectable in the receptor solution for up to 6 h post‐application. Trace amounts were first detected at 8 h (octocrylene: 8.1 μg/cm^2^, 0.04%; avobenzone: 8 μg/cm^2^, 0.17%), with cumulative amounts reaching 264 μg/cm^2^ (1.44%) and 41 μg/cm^2^ (0.90%) respectively by 24 h. In contrast, the general sunscreen exhibited significant and rapid penetration. Octocrylene (38.9 μg/cm^2^, 0.14%) and avobenzone 18.8 μg/cm^2^ (0.25%) were detected as early as 2 h, with their concentrations increasing progressively over 24 h to 1749 μg/cm^2^ (6.08%) and 316 μg/cm^2^ (4.20%), respectively.

These findings clearly demonstrate that the innovative microencapsulation technology significantly impedes and delays the skin penetration of UV absorbers.

#### 3.3.2 In Vivo Raman Spectroscopy Evaluation

This permeability test uses the characteristic Raman signal that distinguished the sample from the skin intrinsic signal to confirm its distribution in different skin depth spaces. Preceding this analysis, a reference of the intrinsic in vivo human skin Raman spectrum was established, with characteristic peaks attributed to specific molecular constituents (Table 5).

**TABLE 5:** The attribution of Raman characteristic peaks and their representative components.

Peak position (cm^−1^)	Vibration mode	Principal representative component
943	N(C‐C) skeleton, collagen skeleton	Proline, hydroxyproline
1275	C‐N absorption band (amide III band)	Glycine skeleton, proline, nucleic acid
1455	C‐H bending pattern of protein (CH_2_ stretching/CH_3_ asymmetric change shape)	Structural protein, elastin
1655	vC=O stretching vibration (amide I band, containing α‐fold, β‐fold and random crimp)	Actin, collagen, keratin
2846	Asymmetric stretching of CH_2_	Lipid
2882	Symmetric stretching of CH_2_	Lipid
2934	Asymmetric stretching of CH_3_	Lipids and proteins

**FIGURE 2:**
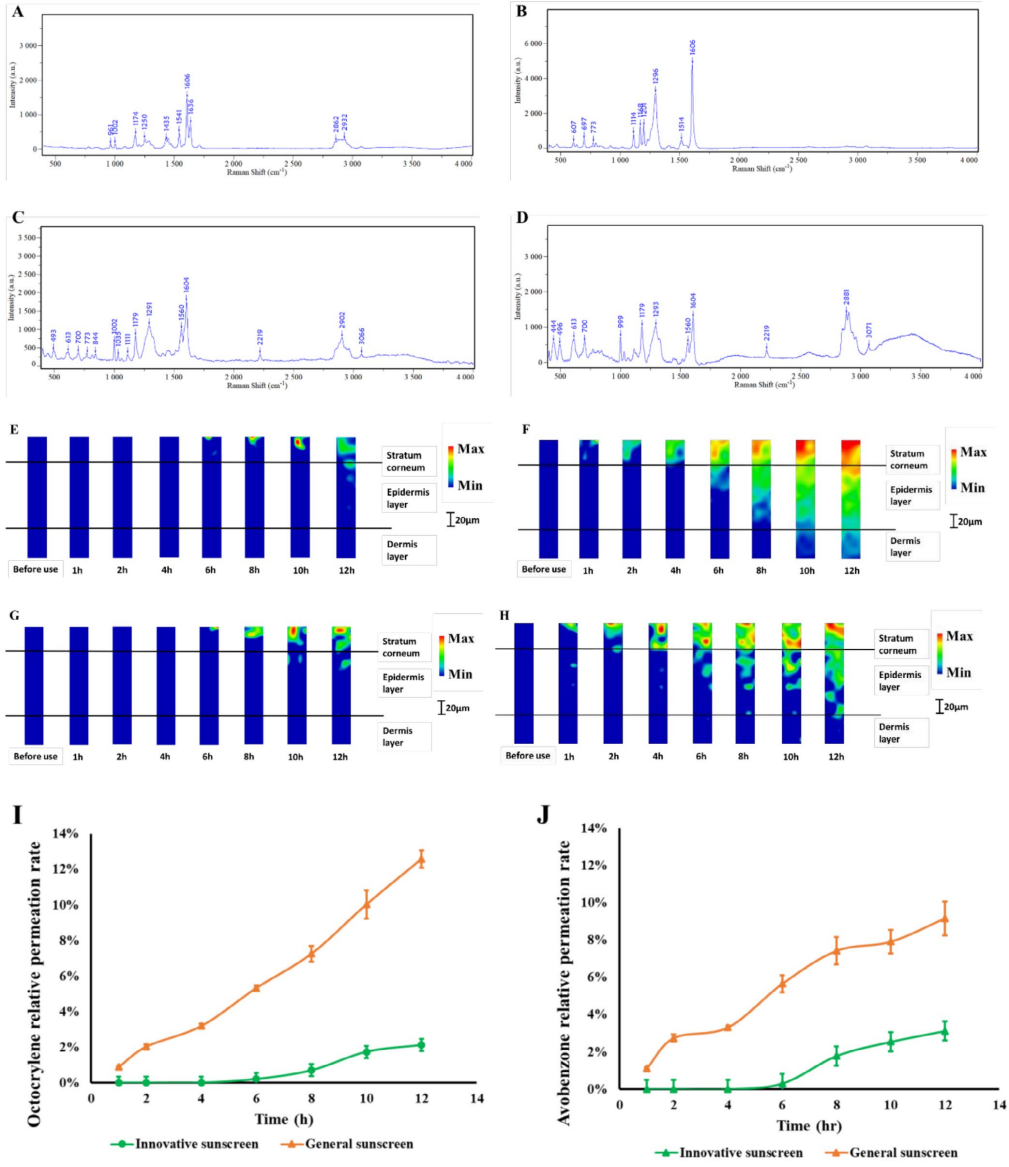
In vivo skin permeation analysis of UV absorbers via confocal Raman spectroscopy. The innovative sunscreen demonstrated significantly reduced skin permeation of octocrylene and avobenzone compared to the general sunscreen. Raman spectra of pure octocrylene (A) and avobenzone (B), the innovative sunscreen (C) and the general sunscreen (D). Depths‐resolved distribution maps of octocrylene in the innovative (E) and general (F) sunscreens, and avobenzone in the innovative (G) and general (H) sunscreens. Cumulative permeation volume of octocrylene (I) and avobenzone (J) in the skin over time for formulations.

The characteristic Raman peaks were identified for pure octocrylene (Figure 2A), pure avobenzone (Figure 2B), as well as for these UV absorbers within the innovative (Figure 2C) and general (Figure 2D) sunscreen formulations.

Depth profiling analysis of the Raman peaks revealed markedly different penetration profiles between the two sunscreen formulations. For the innovative sunscreen, neither octocrylene (Figure 2E) nor avobenzone (Figure 2F) penetrated the stratum corneum (SC) within the first 4 h. Only minimal penetration into the SC was observed between 6 and 10 h, with a slight breach into the viable epidermis detected solely at the 12 h time point. In stark contrast, both UV absorbers from the general sunscreen penetrated the SC within 1 h (Figure 2G,H) and continued to accumulate in the SC and viable epidermis over time. Notably, octocrylene had reached the dermis by 10 h, followed by avobenzone within 12 h.

The accumulated penetration volumes of octocrylene (Figure 2I) and avobenzone (Figure 2J) unequivocally demonstrated that the innovative technology effectively prevented penetration into the SC for up to 4 h. This was further supported by the relative permeation rates, which remained low for the innovative formulation throughout the 12‐h period. The maximum rates observed at 12 h were merely 2.12% for octocrylene and 3.13% for avobenzone. Conversely, the general sunscreen exhibited substantially higher and steadily increasing permeation rates, which exceeded 12% for octocrylene and 9% for avobenzone by the end of the experiment.

### 3.4 In Vivo Clinical Tests

#### 3.4.1 Sensitive Skin Evaluation

Thirty sensitive skin volunteers joined and finished the 4‐week clinical trial. The results were summarized in Table 6. For the innovative sunscreen, only one subject (3.33%) reported mild itching at Day 7, with all other subjects (96.67%) reporting no adverse responses. From Day 14 through Day 28, 100% of subjects reported no adverse reactions. According to the T/GDCA 029—2023 standard (which classifies a product as suitable for sensitive skin if ≥ 96.67% of sensitive skin subjects report no adverse reactions), the innovative sunscreen is confirmed to be mild and suitable for sensitive skin consumers. For the general sunscreen, adverse reactions (itching, redness, burning) were reported by 10% of subjects at Day 7 and persisted throughout the study, with a final nonresponse rate of 96.67% only at Day 28. Consequently, the general sunscreen was classified as less suitable for sensitive skin. These results indicated that the microencapsulation technology significantly reduced the potential for skin irritation.

**TABLE 6:** Sensitive skin volunteers' subjective evaluation results.

Sample	Skin response	Days				Percentage			
		D7	D14	D21	D28	D7 (%)	D14 (%)	D21 (%)	D28 (%)
Innovative sunscreen	No response	29	30	30	30	96.67	100.00	100.00	100.00
	Itch	1	0	0	0	3.33	0.00	0.00	0.00
	Redness	1	0	0	0	3.33	0.00	0.00	0.00
	Burning	0	0	0	0	0.00	0.00	0.00	0.00
	Others	0	0	0	0	0.00	0.00	0.00	0.00
General sunscreen	No response	27	28	28	29	90.00	93.33	93.33	96.67
	Itch	2	1	1	0	6.67	3.33	3.33	0.00
	Redness	1	1	1	1	3.33	3.33	3.33	3.33
	Burning	1	0	0	0	3.33	0.00	0.00	0.00
	Others	0	0	0	0	0.00	0.00	0.00	0.00

#### 3.4.2 Sensory Evaluation

**FIGURE 3:**
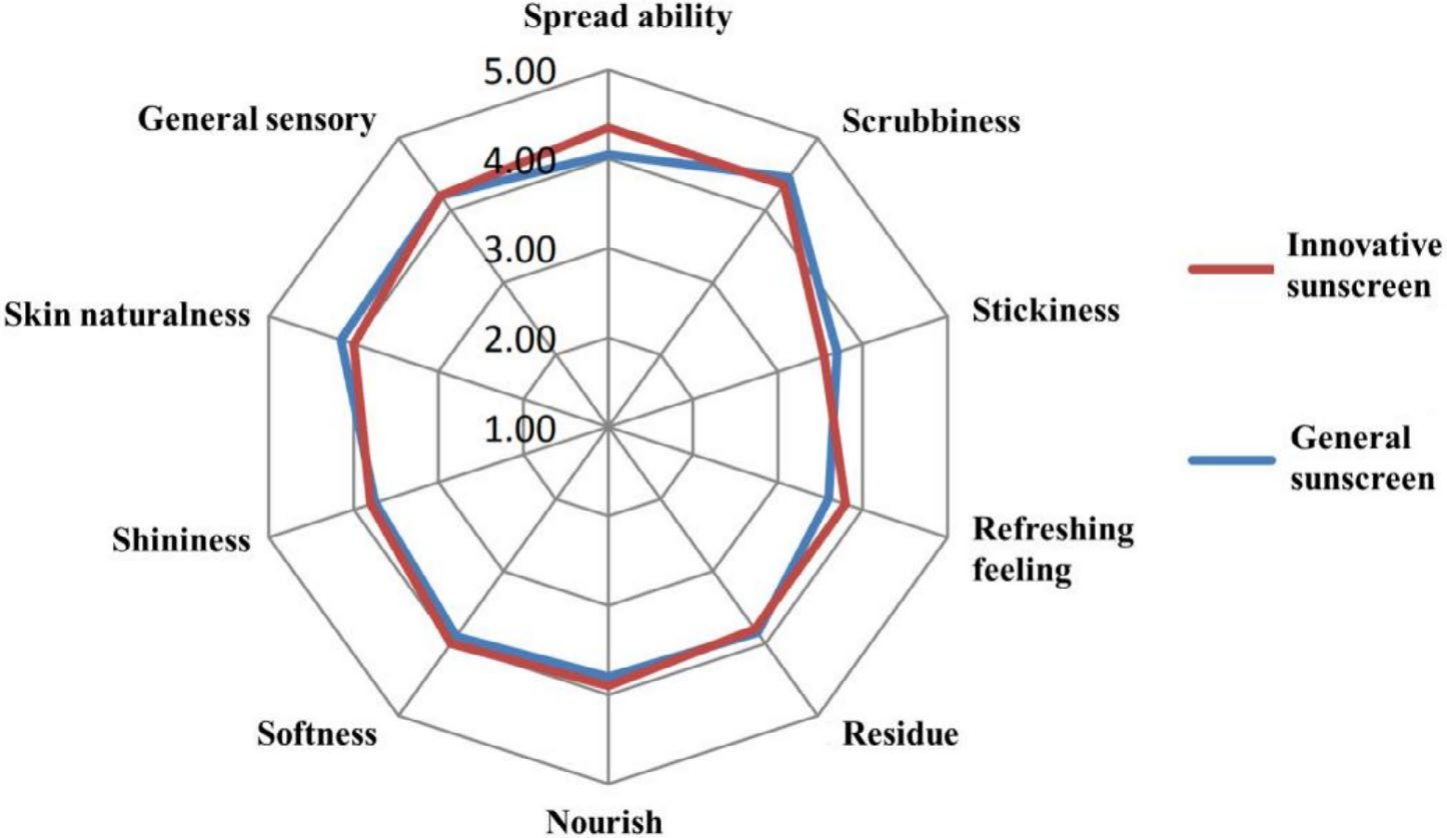
Sensory evaluation map of the innovative and general sunscreens.

All volunteers reported no adverse responses during the sensory evaluation. Statistical analysis of the ratings of ten sensory attributes (e.g., spreadability, stickiness, and refreshing feeling) showed that the data for both sunscreens followed a normal distribution (*p* < 0.05). A comparative analysis revealed no statistically significant difference (*p* > 0.05) in the sensory profile between the innovative and general sunscreens. As illustrated in the sensory profile map (Figure 3), both formulations were rated favorably overall, providing a similarly positive user experience.

## 4 Discussion

A primary function of sunscreens is to remain on the skin surface, forming a protective barrier against UV radiation. However, a significant challenge in formulation is that many UV absorbers can permeate the skin, raising safety concerns due to their potential for systemic absorption and associated risks, such as endocrine disruption and skin irritation [3, 4, 5, 6, 27, 28]. In response, the U.S. Food and Drug Administration (FDA) reclassified 10 UV blockers—including avobenzone and octocrylene—from Category I (Generally Recognized as Safe and Effective, GRASE) to Category III (non‐GRASE), citing insufficient data on their systemic absorption and potential health effects [5]. The FDA has further proposed a plasma concentration safety threshold of 0.5 ng/mL for sunscreen active ingredients [29]. Alarmingly, clinical studies demonstrated that after maximal full‐body application, marketed recreational sunscreens caused all tested UV absorbers to exceed this threshold [6]. Compounding these issues, consumer compliance is often hindered by undesirable product characteristics, including a heavy, oily feel, unnatural whitening, and growing environmental concerns [8].

To address these limitations, this study developed an innovative sunscreen utilizing a polysilicone‐14‐based microencapsulation technology to entrap the UVB absorber octocrylene and the UVA absorber avobenzone. This combination was selected for its proven broad‐spectrum efficacy and photostability [3, 4, 16, 17, 18], while the microencapsulation strategy was specifically designed to mitigate skin penetration [1, 19].

Our comprehensive evaluation, benchmarked against a conventional nonencapsulated sunscreen, demonstrated that the innovative formulation successfully achieved its design goals. The product was confirmed to be nonirritating in the HRIPT study and provided robust in vivo sun protection (SPF ~30, PFA ~10), equivalent to the control. Crucially, the innovative technology dramatically reduced skin penetration. In vitro Franz cell assays showed no detectable permeation of either UV absorbers for up to 6 h. This was corroborated by in vivo Raman spectroscopy, which visually confirmed that the UV absorbers did not breach the SC for 4 h and only minimally penetrated into the viable epidermis after 8–10 h—a stark contrast to the significant and rapid permeation observed with the conventional formula. The superior performance translated directly to enhanced user safety, as evidenced by the clinical trial where the innovative sunscreen was well‐tolerated by volunteers with sensitive skin. These results directly address the major challenges of systemic absorption and photodegradation associated with Category III non‐GRASE UV filters. Our findings are consistent with other encapsulation strategies, such as lignin‐microencapsulated avobenzone for enhanced photostability [13], porous corn starch for reduced penetration [30], and silica or zeolite‐based systems as reviewed by Safian et al. [6]. A preliminary study also indicated that the formulation's residue is easily removed with a general facial cleanser.

Beyond safety and efficacy, the innovative sunscreen also meets key benchmarks for ideal sun protection. The SPF/PFA ratio of less than 3 (30.2/10.4) indicates balanced UVA/UVB protection, which is critical for preventing photoaging and DNA damage [8, 19, 31]. Moreover, the selection of environmentally degradable components—octocrylene [6, 32], avobenzone [5, 12], and silk peptide polysilicone‐14—aligns with the growing demand of eco‐conscious cosmetic products.

In summary, the polysilicone‐14 microencapsulation technology presented here represents a significant advancement in sunscreen formulation. It effectively decouples efficacy from safety by localizing UV absorbers on the skin surface, thereby offering a safer, sensorially pleasant, and environmentally considerate option, particularly suited for consumers with sensitive skin.

## 5 Conclusion

This study comprehensively evaluated a novel sunscreen formulated with polysilicone‐14 microencapsulated UV absorbers octocrylene and avobenzone. The key finding is that this innovative technology effectively prevented the skin penetration of UV absorbers for up to 10 h, without compromising the product's sun protection efficacy (SPF 30, PFA 10) or its positive sensory attributes. The data collectively demonstrated that this microencapsulation strategy successfully addresses the critical industry challenge of minimizing the systemic absorption of topical UV absorbers. Consequently, this technology provides a robust platform for developing a new generation of safer and more effective sunscreens that cater to the needs of the broader population, including individuals with fragile and sensitive skin. The adaptability of this microencapsulation system also holds promise for incorporating other active ingredients, paving the way for future multifunctional skincare products.

## Author Contributions

W.Z. and R.L. performed the research. W.H. and H.K. designed the research study. S.T. contributed essential reagents. X.C. analyzed the data and wrote the paper.

## Ethics Statement

The research protocol was examined and approved by the China‐norm Ethics Committee for Clinical Research (Approval No. GXLL2022006). Benefits, risks, and potential complications were explained to the subjects. All subjects voluntarily participated in this study and signed an informed consent form.

## Conflicts of Interest

The authors declare no conflicts of interest.

## Data Availability

Research data are not shared.
